# Macrophage Colony-stimulating Factor Mediates the Recruitment of Macrophages in Triple negative Breast Cancer

**DOI:** 10.7150/ijbs.39063

**Published:** 2019-11-08

**Authors:** Xiaoqing Lu, Rui Yang, Lichao Zhang, Yanfeng Xi, Jiping Zhao, Fusheng Wang, Huanhu Zhang, Zhuoyu Li

**Affiliations:** 1 Breast surgery, the second Hospital of Shanxi Medical University, Taiyuan 030001, China.982602719@qq.com.; 2 The second Clinical Medical College, Shanxi Medical University, Taiyuan 030001, China.1456236003@qq.com.; 3 Institute of Biomedical Sciences, Shanxi University, Taiyuan 030006, China. zlc@sxu.edu.cn; 4 Department of Pathology, Shanxi Cancer Hospital, Taiyuan 030001, China. xiyanfeng1998@163.com.; 5 Breast surgery, the second Hospital of Shanxi Medical University, Taiyuan 030001, China. 17747001066@189.cn. 13099096632.; 6 Department of Digestive Sciences, Shanxi Cancer Hospital, Taiyuan 030001, China. zhhh31@163.com. 18035119990.; 7 Institute of Biotechnology, Key Laboratory of Chemical Biology and Molecular Engineering of National Ministry of Education, Shanxi University, Taiyuan, 030006, China. lzy@sxu.edu.cn. 13934565188.; 8 School of Life Science, Shanxi University, Taiyuan, 030006, China. lzy@sxu.edu.cn. 13934565188.

**Keywords:** triple negative breast cancer, macrophage migration, macrophage colony-stimulating factor, cytoskeleton, filamentous actin

## Abstract

Triple negative breast cancer (TNBC) is characterized by aggressive malignant tumor, poor prognosis and lack of targeted treatment. Several studies have established that macrophages are closely associated with the progression of TNBC. Through immunohistochemical analysis, we found that the infiltration of macrophage in TNBC tissue was more than that in non-triple negative breast cancer (nTNBC) tissue. Furthermore, the conditioned medium (CM) of MDA-MB-231 and HCC1937, the TNBC cell lines, had significant migration-promoted effect on macrophages. However, the macrophages migration-promoted ability of nTNBC cell line MCF-7 was weaker than that of MDA-MB-231 and HCC1937 cells. Mechanistically, MDA-MB-231 and HCC1937 cells secreted more macrophage colony-stimulating factor (M-CSF) than MCF-7, which is the main inducer of macrophage migration, and the secreted M-CSF promoted the increase in actin and the elongation of pseudopodia. When M-CSF was neutralized by antibody, the elongation of macrophage pseudopodia was disappeared and the migration was inhibited. *In vivo*, there were more macrophages in tumors induced by MDA-MB-231 than MCF-7 did. Therefore, M-CSF specially secreted by TNBC was the important cause of macrophages aggregation in TNBC, which further promoted the aggressiveness of TNBC.

## Introduction

Globally, breast cancer is the most common malignant cancer among women along with increasing incidence rate every year 1. With the technological progress of the early diagnosis of breast cancer, there is a gradual progress in the comprehensive treatment strategies for breast cancer, which include radiotherapy, chemotherapy, and biological therapy 2-3. Breast cancer is genetically heterogeneous and can be divided into four subtypes, which have different molecular pathology and require different therapeutic strategies. The four subtypes of breast cancer are Luminal A, Luminal B, Her-2 overexpression and triple negative subtypes. Among the four subtypes, triple negative breast cancer (TNBC) is more prevalent among premenopausal young women, with higher cell proliferation rate, poor histopathological grade, and higher visceral metastasis. In contrast to the other three non-triple negative breast cancers (nTNBC), TNBC lacks a specific treatment plan. Additionally, the endocrine therapy and targeted therapy are ineffective in treating TNBC 4.

Several studies have indicated that the occurrence and development of tumors are not only due to the genetic heterogeneity of tumor cells, but also closely related to the tumor-microenvironment (TME) 5. During the growth of solid tumors, cancer cells interact with the TME. The components of TME modulate the progression of tumor by providing cancer cells with stimulatory or inhibitory signals. The cancer cells also release several signaling molecules to regulate the components of the microenvironment, making the microenvironment more conducive for the growth, invasion, or metastasis of cancer cells 6-7.

Macrophage is an important component of innate immunity and form the primary component of the mononuclear phagocyte system, which maintains the physiological homeostasis and responds to the pathogenic invasion 8. Macrophages exhibit strong plasticity and have distinct functions in various tissue components 9. In the TME, macrophages that are recruited around the tumor are activated to form the tumor-associated macrophages (TAMs), which are the most abundant mononuclear cells in the tumor infiltrating leukocytes 10. Many studies have shown that TAMs are associated with poor prognosis of tumors 11-12. Particularly, the density of TAMs is closely associated with the poor prognosis of some solid tumors, such as breast cancer, gastric cancer, lung cancer, and bladder cancer 13-14. Thus, the recruitment of macrophages to the TME is important before the transformation to TAMs.

In this study, we observed that the TNBC cells recruited more macrophages than the nTNBC cell by secreting more macrophage colony-stimulating factor (M-CSF). As macrophages have a role in the tumor progression, our study will provide a new strategy for TNBC treatment by targeting M-CSF.

## Materials and Methods

### Immunohistochemistry

54 paraffin-embedded breast cancer tumor tissue blocks were obtained from the Department of Pathology, Shanxi Cancer Hospital (Shanxi, China). The detailed clinicopathological information for all the enrolled patients was available. For immunohistochemical analysis, 54 paraffin-embedded tissues were cut into 3 μm serial sections. The tissue specimens were subjected to immunohistochemical analysis following standard procedures. Anti-estrogen receptor (ER) (Gene Tech, Shanghai, China) and anti-CD68 antibodies (Maxim, Fujian, China) were used for immunostaining. The images were captured under a microscope fitted with a charge-coupled device (CCD) camera. The immunohistochemical score was assigned to each specimen according to the staining intensity of the membrane, cytoplasm, and nuclei (no staining = 0; weak staining = 1; moderate staining = 2; strong staining = 3) and the percentage of positive cells (0% = 0; 1-24% = 1; 25-49% = 2; 50-74% = 3; 75-100% = 4). A final score was obtained by multiplying the intensity score with the percentage score of the stained cells, which ranged from 0 (the minimum score) to 12 (the maximum score). The integrated optical density (IOD) value of immunostaining was measured using Image-Pro Plus 6.0(Media Cybernetics Corporation, USA).

### Xenograft mice

Female BABL/c nude mice (4-week-old, n = 20) were purchased from China Institute for Radiation Protection (Peking, China). All animal experiments conducted in this study were approved by the Experimental Animal Center, Shanxi Cancer Hospital (Taiyuan, China). 20 immunodeficient (nu/nu) mice were randomly divided into two groups (*n* = 10). Equal amounts (2 × 10^6^) of MCF-7 cells or MDA-MB-231 cells were re-suspended in 200 μL of PBS buffer and injected into the subcutis of mice. Then mice were euthanized when the tumor grew to between 1.5 and 2 cubic centimeters, tumors were excised and analyzed by immunohistochemistry.

### Cell lines and cell culture

In this study we used three breast cancer cell lines, MCF-7, MDA-MB-231, HCC1937 and a macrophage cell line RAW264.7. The MCF-7 and RAW264.7 cell lines were available at our lab. The MDA-MB-231 cell line was obtained from EnoGene Biotech Co. Ltd (Nanjing, China). The HCC1937 cell line was obtained from Shanghai Zhong Qiao Xin Zhou Biotechnology Co., Ltd (Shanghai, China). The MCF-7 and HCC1937 cells were grown in Roswell Park Memorial Institute-1640 (RPMI-1640, Biological Industries, Israel Beit-Haemek) medium, while the RAW264.7 and MDA-MB-231 cells were grown in Dulbecco^'^s modified Eagle^'^s medium (DMEM, Biological Industries, Beit-Haemek, Israel), supplemented with 10% (v/v) fetal bovine serum (FBS, Tianhang Biotechnology, Zhejiang, China) and 1% (v/v) penicillin-streptomycin (Solarbio, Peking, China) solution at 37 °C in a humidified tissue culture incubator containing 5% CO_2_.

### Collection of conditioned medium

The three breast cancer cells were grown till the exponential phase and seeded in 10.0 cm culture dishes at equal density. When the cells were attached to the culture dish and attained a confluency of 70-80%, the growth medium was replaced with 10 mL fresh medium and incubated for 24 h. Further, the three breast cancer cells conditioned medium (CM) were obtained by centrifuging at 3000 *g* for 15 min. The macrophage CM was obtained by seeding the exponential phase macrophages in a 10.0 cm culture dish. The cell culture medium was aspirated when the cells were attached to the culture dish and attained 50-60% confluence. Then the macrophages were treated with 20% (in DMEM) breast cancer cell CM. The culture medium was replaced after 48 h of incubation, and the cells were cultured for another 24 h. The culture medium was centrifuged and the supernatant was collected as the macrophage CM.

### Transwell migration assay

Transwell chambers (Jet Bio, Guangzhou, China) were placed in a 24-well culture plate. The cell number of macrophages in the exponential phase was adjusted to 1 × 10^6^ cells/mL using DMEM medium supplemented with 1% (v/v) FBS. The cells (100 μL) were inoculated into the upper Transwell chamber. To the lower chamber, the breast cancer cells CM were added at concentrations (v/v) of 0, 10, 20, 40, 80 and 100% to fresh DMEM supplemented with 10% FBS. The cells were incubated for 48 h and that in the upper layer of the filter were wiped using a cotton swab. The filter was fixed with 4% paraformaldehyde for 10 min and was stained with 0.1% crystal violet dye (Solarbio, Peking, China) for 15 min. Five different fields of view were randomly selected for imaging under a 10× objective of the light confocal microscope (Zeiss, Oberkochen, Germany). The average number of cells that passed through the membrane was calculated using Image J v1.8.0 software.

### Wound healing assay

Before inoculation of the macrophages in the culture plate, a horizontal line was marked on the back of the 12-well plate with a marker pen (to locate the same field of view while imaging). The macrophages were seeded in the 12-well plate and allowed to form a monolayer. Scratches were made perpendicular to the well plate using a 200 μL tip and the width of each scratch was as uniform as possible. The cell culture medium was removed and the cells were rinsed three times with phosphate buffer saline (PBS, PH=7.4) to remove the cell debris generated by the scratch. The macrophages were treated with the MCF-7 cell CM(M-CM), MDA-MB-231 cell CM(MDA-CM), or HCC1937 cell CM(H-CM) respectively at a concentration of 20% (v/v) in DMEM supplemented with 1% FBS. An equal volume of fresh DMEM supplemented with 1% FBS was used as a control. The culture plate was incubated and imaged at 0, 12, 24, and 48 h. The scratch area was measured using Image J v1.8.0 software and the percentage of wound healing was calculated according to the following formula: Percentage of wound healing = [(scratch area at 0 h - scratch area at the indicated time point)/ (scratch area at 0 h)] × 100.

### MTT assay

The macrophages were collected at the exponential phase and seeded into a 96-well plate (the edge wells were filled with sterile PBS). The culture medium in the well was aspirated, and the cells were treated with the CM of breast cancer cells. The assay was performed with 6 replicates. After incubation for 48 h, the culture medium was replaced with 200 μL fresh DMEM. Next, 20 μL 3-[4,5-dimethylthiazole-2-yl]-2,5-diphenyltetrazolium bromide (MTT) solution (Solarbio, Peking, China) was added to each well. Further, 150 μL DMSO was added to each well after 4 h incubation, and incubated on a shaker at low speed for 5 min to dissolve the crystals sufficiently. The absorbance of each well was measured using a microplate reader at 570 nm. The mean optical density (OD) was calculated from the duplicate wells and the cell proliferation rate was calculated according to the following formula: Cell proliferation rate = [(treatment group OD value - control group OD value) / (control group OD value)] × 100.

### Gelatin zymography

The CM of macrophages was analyzed for gelatin degradation by electrophoresis using sodium dodecyl-polyacrylamide gel containing 1 mg/mL gelatin. The volume of CM loaded per lane was standardized on the basis of the cell count. The gels were incubated overnight at room temperature (25 °C) in 50 mm Tris-HCl, 150 mm NaCl, and 10 mm CaCl_2_ (pH 7.4). The white lysis zone, which indicated the gelatin degradation, was identified by staining with Coomassie brilliant blue. The blots were documented using Canon CanoScan 9000F Mark II scanner (Canon, Peking, China).

### Cell adhesion assay

The macrophages, which were treated with breast cancer cell CM for 48 h, were harvested and counted. The cells were then seeded in 96-well plate at a density of 3000 cells per well. Immediately after inoculation, the macrophages in the six replicate wells were discarded every two minutes. At the end of the final washing step, MTT was added to the wells. The absorbance was measured as described previously in MTT assay. The time at which maximum OD value was obtained was regarded as the time when all the macrophages adhered to the bottom. The percentage adhesion for the macrophages was calculated according to the following formula: Percentage adhesion = [(the OD value of a certain time - OD value of 0 min) / (max OD value - OD value of 0 min)] × 100.

### Immunofluorescence

Actin staining was performed using phalloidin (Acti-stain™ Fluorescent Phalloidins, Cytoskeleton, Inc., Denver, USA) following the manufacturer's instructions. The macrophages were seeded into 12-well glass slides and treated with breast cancer cell CM. The cells were then fixed in 4% paraformaldehyde in PBS for 30 min. Next, 200 μL 100 nM Acti-stain™ 488 phalloidin and 100 nM DAPI solution in PBS were added. The cells were incubated at room temperature (25 °C) in the dark for 30 min. The cells were washed three times using PBS. The slides were mounted for confocal immunofluorescence analysis.

### Flow Cytometry

Macrophages, which were treated with breast cancer cell CM for 48 h were harvested and fixed in 70% ethanol for 30 min. The cells were subjected to actin staining using phalloidin and incubated at room temperature (25 °C) in the dark for 30 min. The cells were resuspended using PBS and subjected to flow cytometry. Flow cytometry results were analyzed using FlowJo software (FlowJo, LLC, Ashland, USA).

### Quantitative PCR

The total RNA was extracted using Trizol reagent and reverse-transcribed into complementary DNA (cDNA) for quantitative polymerase chain reaction (qPCR) following the manufacturer's instructions. *Gapdh* gene served as an endogenous control. The relative expression of each targeted gene was normalized by subtracting the corresponding *Gapdh* threshold cycle (Ct) values using the ΔΔCt method. The primers used in this study are listed in Supplementary Table 1.

### Western blotting

The breast cancer cells were lysed in RIPA lysis buffer (Solarbio, Peking, China) containing a protease inhibitor cocktail (Solarbio, Peking, China). The total protein in the supernatant was quantified using a bicinchoninic acid (BCA) protein assay kit (Beyotime, China). The proteins were extracted and resolved by sodium dodecyl sulfate polyacrylamide gel electrophoresis (SDS-PAGE) using 10% gel. The proteins were then transferred to a polyvinylidene fluoride membrane. The membrane was incubated in a solution containing primary antibodies against M-CSF and GAPDH (Proteintech, Wuhan, China) overnight at 4 °C. The protein bands were visualized using radiographic film exposure. The blots were documented using Canon CanoScan 9000F Mark II scanner (Canon, Peking, China).

### Enzyme-linked immunosorbent assay

The enzyme-linked immunosorbent assay (ELISA) was performed according to the manufacturer's instructions (M-CSF ELISA kit, westang, Shanghai, China). The OD of all samples was subtracted from that of the blank. The standard concentration of M-CSF was 400, 200, 100, 50, 25, 12.5, 6.25, and 0 ng/mL. A standard graph was plotted using the concentration as the abscissa and the OD value as the ordinate. The level of M-CSF was measured from the standard curve.

### Pathway enrichment analysis

We selected the top 3234 differential genes from GEO2R results of GSE27473 for pathway enrichment. The list of differentially expressed genes was entered into the Enrichr website. In the output interface, we used Ontologies to select the output of the GO Biological Process 2018 as the analysis results. Finally, the data and images were saved for further analysis.

### Statistical analysis

Statistical analyses were performed using SPSS 20.0 (SPSS Inc, Chicago, IL, USA), and Graphpad Prism 6 (GraphPad Software, Inc., La Jolla, USA). The correlation between ER expression and CD68 expression was analyzed by Pearson's correlation. The data were represented as the mean ± standard error of mean (SEM). Statistical significance among the groups was tested using one-way analysis of variance (ANOVA), The comparison between two groups was evaluated using Student's *t*-test. The data was considered statistically significant when the P value was less than 0.05.

## Results

### The infiltration of macrophages was more in TNBC than that in nTNBC

**Figure 1:**
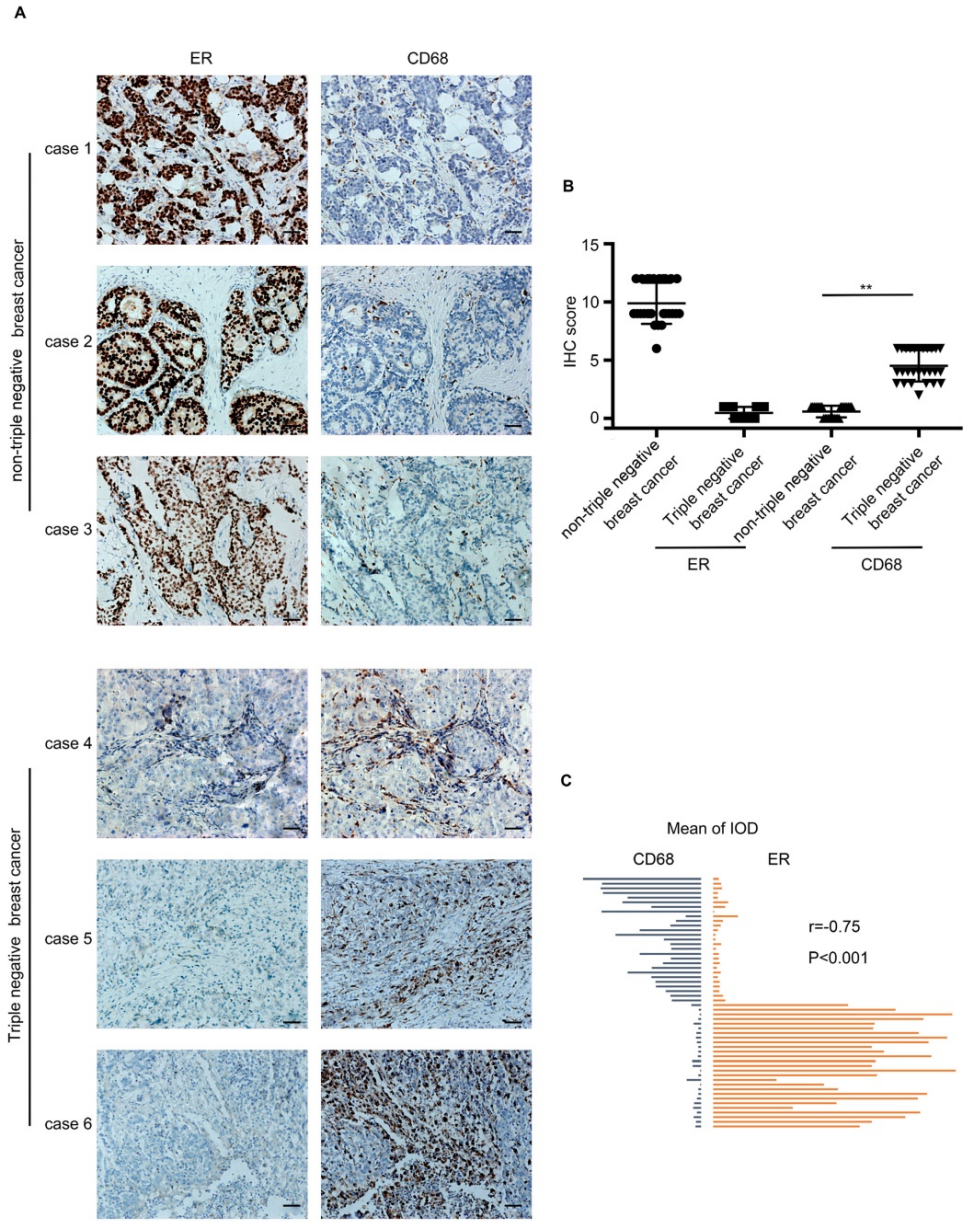
The infiltration of macrophages in the triple-negative breast cancer cells (TNBC) and the non-triple-negative breast cancer (nTNBC). ** (A)** The detection of ER and CD68 in paraffin-embedded breast cancer specimens. Brown staining denotes the immunoreactivity of ER or CD68. The tumor sections were counterstained by Hematoxylin to label the nuclei. Scale bar, 50 μm. **(B)** Box plot analysis revealed higher CD68 immunohistochemistry (IHC) score in the TNBC cells. **P<0.001. Bars represent the mean ± standard deviation (SD). **(C)** Pearson's correlation analysis showed a negative correlation between ER integrated optical density (IOD) and CD68 IOD.

54 breast cancer specimens were collected, which included 27 TNBC cases and 27 nTNBC cases. The specimens were detected for two markers, ER and CD68 (macrophage marker). As shown in Figure 1A, the infiltration of macrophages in the TNBC specimen was higher than that in the nTNBC specimen, and the IHC score of CD68 was different significantly (P<0.001) between the two cancer types (Figure 1B). Moreover, Pearson's correlation analysis revealed that there was a significant negative correlation between the expression of CD68 and ER in the breast cancer (r=-0.75, P<0.001) (Figure 1C). These *in vivo* data indicated that the TNBC was more attractive than nTNBC for macrophages, and the ER maybe play an important role in this process.

### TNBC cells promoted the migration of macrophages

**Figure 2:**
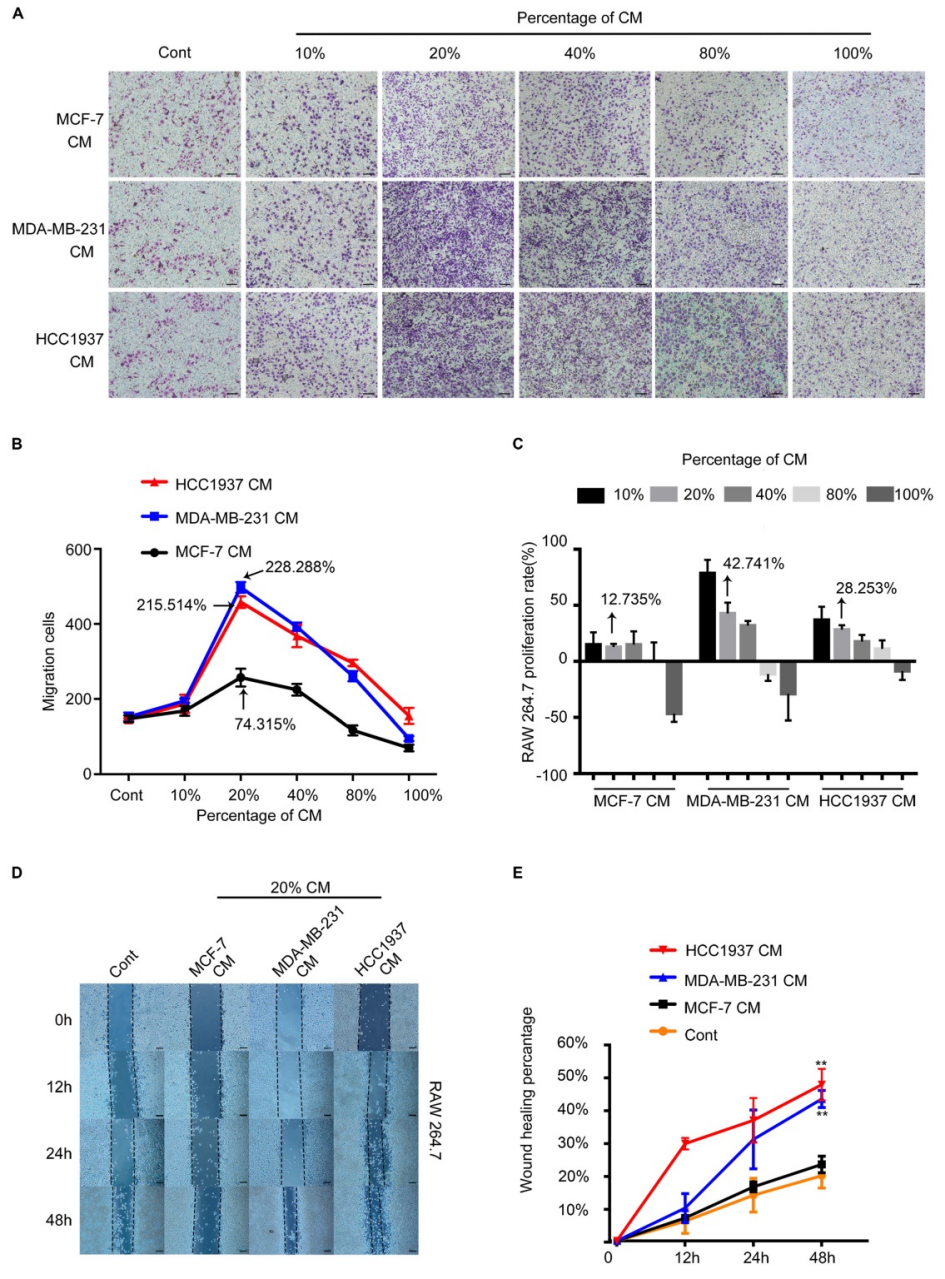
The RAW264.7 cell recruitment capacity of the MCF-7, MDA-MB-231, and HCC1937 cells *in vitro*. ** (A)** The migration of macrophages in different concentrations of breast cancer cell conditioned medium (CM). The control group was the fresh medium without CM. Scale bar, 100 μm. **(B)** The difference in the number of macrophages recruited upon treatment with different concentrations of CM was compared using one-way ANOVA, which was significantly different. P<0.001. The pro-migration effect of the three different conditioned media on macrophages was significant. P=0.0322. At a concentration of 20%, CM exhibited the highest pro-migration effect on macrophages. P<0.001. **(C)** MTT assay demonstrated that the proliferation rates of the M-CM, MDA-CM and H-CM groups were 12.735%,42.741% and 28.253%, respectively. **(D, E)** At 20% concentration, the wound healing assay demonstrated that the migration promoting effect of triple-negative breast cancer (TNBC) cells on macrophages was significantly higher than that of MCF-7 cells. P<0.001.

To test the ability of breast cancer cells recruiting macrophages, the conditioned medium (CM) of breast cancer cells were collected to test their migration-promoted effect by Transwell migration assay. As shown in Figure 2A, the MDA-MB-231 CM(MDA-CM) or HCC1937 CM(H-CM) dramatically facilitated the migration of macrophages. However, the migration-promoted effect of MCF-7 CM(M-CM) was weaker than that of MDA-CM and H-CM. In addition. It was clear that macrophages which were treated with 20% CM of breast cancer cells had the strongest migration ability than other CM concentrations, and the pro-migration rates of the M-CM, MDA-CM and H-CM groups were 74.315%,215.514% and 228.288%, respectively. (Figure 2B, P<0.001). We further evaluated the proliferation effects of breast cancer cell CM-treated macrophages by MTT assay. The proliferation rates of the M-CM, MDA-CM and H-CM groups were 12.735%, 42.741% and 28.253%, respectively. Above results showed that the migration-promoted ability was stronger than the proliferation-promoted ability of breast cancer cells CM on macrophages (Figure 2C). Furthermore, 20% breast cancer cells CM was the most appropriate concentration for subsequent experiments. The wound healing data was shown in Figure 2D and 2E. After 48 h treatments, MDA-CM-treated and H-CM-treated macrophages exhibited an enhanced migration rate (Figure 2D), and the healing area of scratch was nearly 60% at 48 h in MDA-CM and H-CM-treated groups, which was significantly higher than M-CM-treated macrophages (Figure 2E, P<0.001). All of these results suggested that the macrophages were sensitive to the secretion of TNBC specially.

### TNBC cells CM-treated macrophages exhibited cytoskeletal alterations

**Figure 3:**
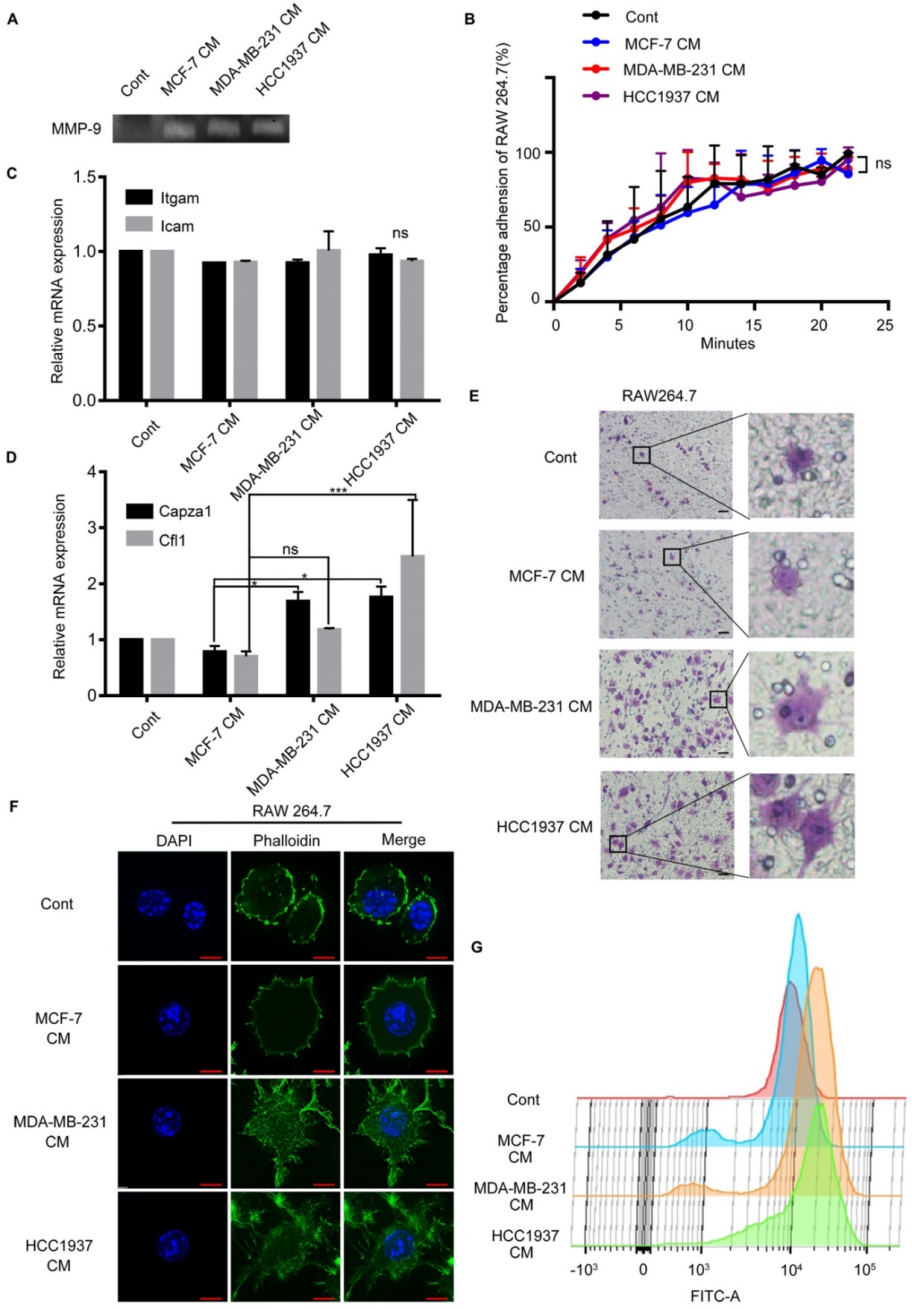
Triple-negative breast cancer (TNBC) cells can alter the cytoskeleton of macrophages. ** (A)** White lysis zone indicates MMP-9 activity in gelatin zymography. **(B)** The adhesion ability of macrophages treated with different CM were not significant. P>0.05. **(C)** Quantitative polymerase chain reaction (qPCR) data showing the mRNA expression of* Itgam* and *Icam* in macrophages treated with different CM. P>0.05. **(D)** qPCR data showing the mRNA expression of *Capza1* and *Cfl1* in macrophages treated with different CM. *P<0.05. **(E)** The cells that passed through the membrane in the Transwell migratory assay were imaged using a confocal microscope. Scale bar, 50 μm. **(F)** Green staining indicates F-actin and blue indicates nuclei in immunofluorescence staining. Scale bar, 10 μm. **(G)** The height of the peak represents the amount of F-actin in the macrophages.

In order to explore the changes occurred in macrophages with the stimulation of TNBC CM; we conducted a series of experiments. Gelatin zymography assay demonstrated that the breast cancer (MCF-7, MDA-MB-231 and HCC1937) cell CM-treated macrophages secreted more matrix metalloprotein-9 (MMP-9) than the untreated group, but there was no difference among the three CM-treated groups (Figure 3A). Enhance of adhesion ability was an important characteristic of cell migration. So, the adhesion ability of macrophages treated by breast cancer cells CM was detected. The results exhibited that the adhesion ability of macrophages was not significantly different in the three groups either. (Figure 3B, P>0.05). Furthermore, we measured the mRNA expression of two classical cell adhesion molecules, intercellular cell adhesion molecule (ICAM) and integrin alpha M (ITGAM), and there was no difference in the *Icam* and *Itgam* expression (Figure 3C, P>0.05).

Interestingly, we observed that the macrophages treated by TNBC CM and crossing the Transwell membrane had a significant morphology change, from round or elliptical shape to long fusiform or polygonal shape obviously (Figure 3E). These phenomena implied that the changes of cytoskeleton were the main cause of the macrophage migration. As widely approved, filamentous actin (F-actin) was regarded as a marker to evaluate the changes in the cytoskeleton for its principal component of the cytoskeleton. The mRNA expression level of *Capza1* and *Cfl1* genes, which represented actin filament and cofilin 1, were examined respectively in the macrophages. The expression of *Capza1* in the H-CM or MDA-CM-treated macrophages was significantly higher than that observed in M-CM-treated macrophages (Figure 3D, P=0.0251, P=0.0401). In order to illustrated the change in macrophage cytoskeleton intuitively, the phalloidin stain was applied to assess the effect of breast cancer cell CM on the actin cytoskeleton of macrophages. After H-CM or MDA-CM treatment, the actin filamentous shape of macrophages was extended, forming larger and brighter amorphous F-actin clusters, and the morphology of these macrophages changed from oval to polygonal or irregular (Figure 3F). Flow cytometry demonstrated that the peak representing the fluorescence intensity of the TNBC cell CM-treated macrophages moved to the right as comparing the untreated or M-CM-treated macrophages (Figure 3G). These results indicated that the TNBC cells increased deformation of macrophages, and further promoted the migration of macrophages.

### More M-CSF was secreted by TNBC than nTNBC

**Figure 4:**
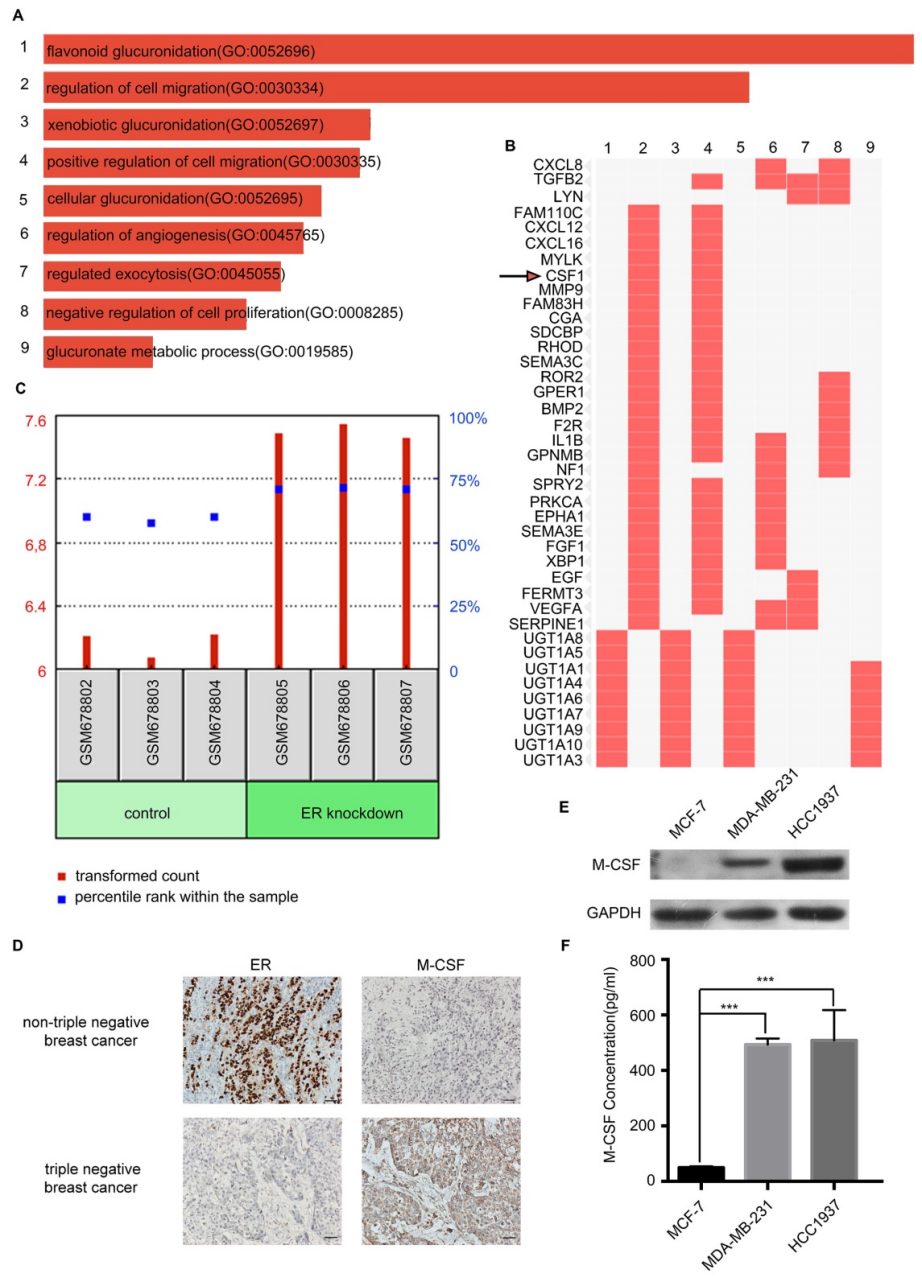
Detection of macrophage colony-stimulating factor (M-CSF) expression and secretion in different breast cancer cells. ** (A)** These bars were sorted by combined score which is computed by taking the log of the P-value from the Fisher exact test and multiplying that by the Z-score of the deviation from the expected rank. **(B)** Enriched biological processes are the columns, input genes are the rows, and cells in the matrix indicate if a gene is associated with a process. **(C)** The GDS4061 dataset showed that the expression of M-CSF in the ER knockout MCF-7 cells was significantly higher than that in the control. P<0.05. **(D)** The detection of ER and M-CSF in paraffin-embedded breast cancer specimens. Brown staining denotes the immunoreactivity of ER or M-CSF. The tumor sections were counterstained by Hematoxylin to label the nuclei. Scale bar, 50 µm. **(E)** Western blotting revealed that the M-CSF level in the HCC1937 cell or MDA-MB-231 cell was higher than that in the MCF-7 cell. **(F)** ELISA showed that the secreted M-CSF level in the HCC1937 CM or MDA-MB-231 CM was higher than that in the MCF-7 CM.

To further distinguish the inducer of macrophage migration from TNBC secretion, the Gene Expression Omnibus (GEO) database of the National Center for Biotechnology Information was analyzed. Interestingly, there was a correlation between ER expression and M-CSF expression in the breast cancer cells. Then the GSE27473 dataset was found by retrieving GEO dataset browser, which was an analysis of MCF-7 breast cancer cells with the estrogen receptor α (ERα) silencing. The ER was silenced in the MCF-7 cell line for RNA extraction and hybridization on Affymetrix expression microarrays. Further, the GSE27473 dataset was analyzed with GEO2R by dividing the data into two groups: control and ER knockdown. The differentially expressed genes were compared between the two groups and the pathway enrichment analyses were performed. After pathway enrichment of differential genes, nine biological processes were screened, among which the second was the regulation of cell migration (Figure 4A). From the cluster figure of the differential genes, we found that the top five genes in the biological process of regulation of cell migration were FAM110C, CXCL12, CXCL16, MYLK, and CSF1. The expression of FAM110C, CXCL12 and CXCL16 in the ER knockdown group was low, while the expression of MYLK and CSF1 was high (Figure 4B). Among MYLK and CSF1, CSF1 (macrophage colony-stimulating factor or M-CSF) is more closely related to the macrophages.

M-CSF is a secreted cytokine that influences the hematopoietic stem cells to differentiate into macrophages or other related cell types. M-CSF is also involved in the proliferation, differentiation, and survival of monocytes, macrophages, and bone marrow progenitor cells. The GDS4061 dataset revealed that the expression of M-CSF in the ER knockout MCF-7 cells was significantly higher than that in the control group (P<0.001, Figure 4C). For immunohistochemical analysis of 54 paraffin-embedded breast cancer patient tissues, M-CSF was low expressed when ER was highly expressed in nTNBC. When there was a low expression level of ER in TNBC, M-CSF expression level was high (Figure 4D).

Western blotting and ELISA analyses revealed that the expression and secretion of M-CSF in the HCC1937 cell or MDA-MB-231 cell was higher than that in the MCF-7 cell (Figure 4E, F). Taken together, there was a remarkable outcome in comparing M-CSF secretion level between TNBC and nTNBC cells, and this indicated that M-CSF may be involved in the recruitment of macrophages in breast cancer.

### M-CSF played a key role in the macrophage recruitment

**Figure 5:**
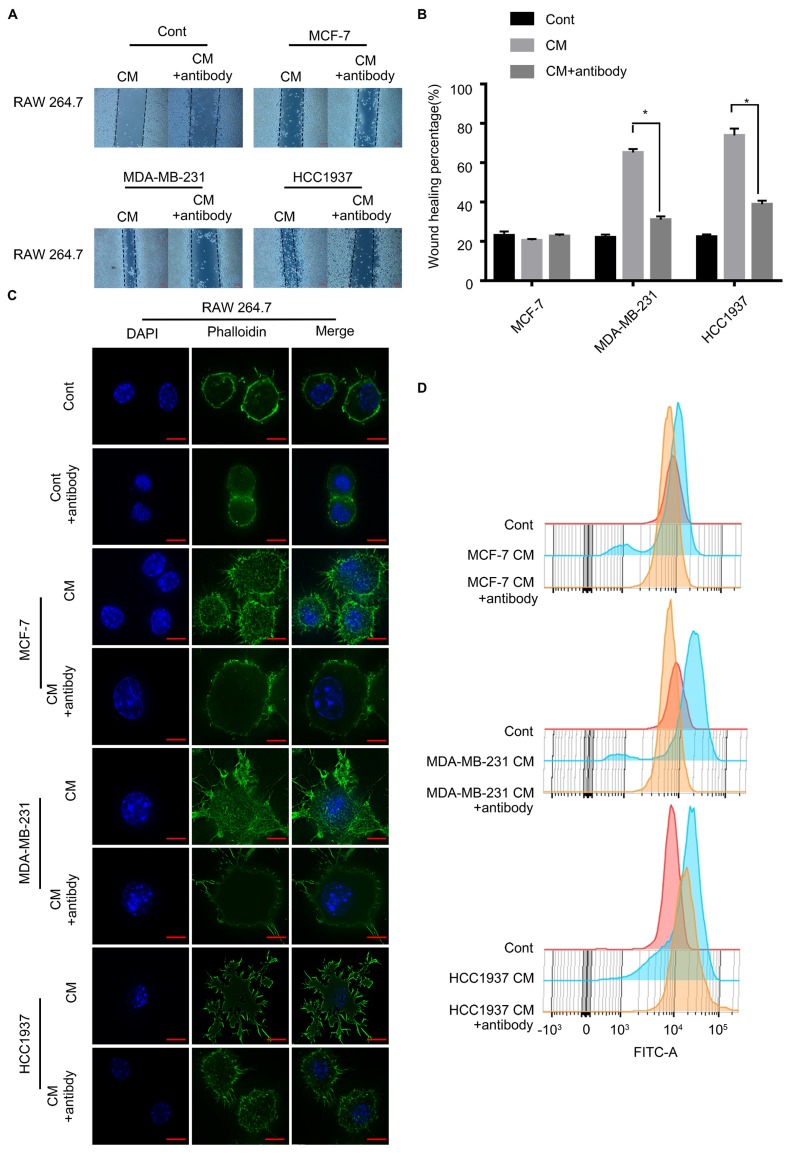
Inhibition of M-CSF can aggravate the recruitment of macrophages to the triple-negative breast cancer (TNBC) cells. **(A, B)** Wound healing assay showed that the ability of scratch healing was significantly smaller after adding M-CSF antibody to the TNBC cell conditioned medium (CM) than that in which the M-CSF antibody was not added. P<0.05. **(C, D)** Adding M-CSF antibodies to CM led to a decrease in the cytoskeletal deformity of macrophages, while the content of F-actin was reduced. Scale bar, 10 µm.

**Figure 6:**
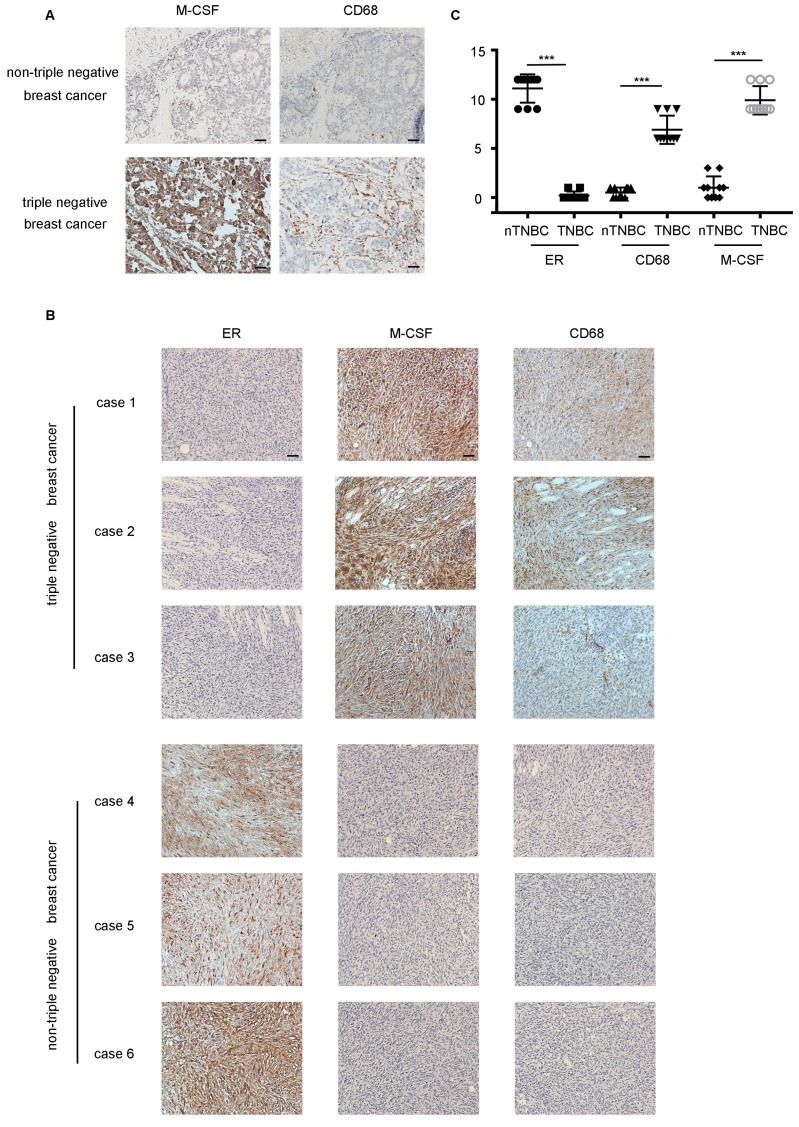
M-CSF played a key role in macrophages recruitment and the infiltration of macrophages was higher in TNBC *in vivo*. ** (A)** The detection of M-CSF and CD68 in paraffin-embedded breast cancer specimens. Brown staining denotes the immunoreactivity of M-CSF or CD68. The tumor sections were counterstained by Hematoxylin to label the nuclei. Scale bar, 50 µm. **(B)** Box plot analysis revealed higher CD68 and M-CSF immunohistochemistry (IHC) score in the xenograft TNBC tumor. ***P<0.0001. Bars represent the mean ± standard deviation (SD). **(C)** The detection of ER, M-CSF and CD68 in immunodeficient mice xenografts. Brown staining denotes the immunoreactivity of ER, M-CSF or CD68. Scale bar, 50 µm.

To test the critical role of M-CSF in macrophage recruitment, we added a polyclonal antibody of M-CSF to the CM to neutralized the M-CSF. In wound healing assay, the macrophage migration promoting effect of the TNBC cell CM was significantly inhibited by the M-CSF antibody (Figure 5A, B, P<0.05). Additionally, M-CSF antibody treatment also inhibited the change of macrophage morphology (from circular to polygonal) by TNBC cell CM treatment (Figure 5C). The increase of F-actin content, observed in the TNBC cell CM-treated macrophages, was also reversed by M-CSF antibody treatment (Figure 5D). For immunohistochemical analysis of breast cancer patient tissues, the higher the expression of M-CSF, the stronger the aggregation of macrophages was found (Figure 6A). These results suggested that M-CSF was a key factor for the macrophage recruitment by TNBC cells.

### *In vivo*, the infiltration of macrophages was higher in TNBC by high expression level of M-CSF

The difference of TNBC and nTNBC on macrophage recruitment was further evaluated in xenografts. The number of CD68+ macrophages was much higher in the mice tumor tissues inoculated with the MDA-MB-231 cells than mice transplanted with the MCF-7 cells (Figure 6B). Meanwhile, mice which were transplanted with the MCF-7 cells showed lower expression of M-CSF in the tumor tissues than that with MDA-MB-231(Figure 6B). These results not only confirmed that macrophage infiltration was more pronounced in TNBC, but also verified the importance of M-CSF in this process simultaneously.

## Discussion

Our studies found that the TNBC cells recruited more macrophages than nTNBC cells, and the secreted M-CSF played a key role in this process. However, why did TNBC cells secrete more M-CSF? In recent years, the relationship between breast cancer and Kindlin-2 expression has been reported. Kindlin-2 belongs to the Kindlin family, which is composed of three members, Kindlin-1, -2 and -3^15^-^16^, of which the Kindlin-2 is involved in multiple diseases, including kidney fibrosis and tumor progression 17. Kindlin-2 protein was significantly upregulated during the emergence of mammary tumors, especially; the Kindlin-2 expression was greatly increased upon in ER-negative mouse mammary tumors 18. Furthermore, Sossey-Alaoui et al. observed that the loss of M-CSF appeared to be caused by a more proximal deficiency in TGFβ-dependent signaling in the Kindlin-2-deficient cells 19. It is concluded that high-expressed Kindlin-2 in ER-negative breast cancer is directly correlated with overexpression of M-CSF, which may be the important course of M-CSF high secretion of TNBC cells in our study.

Macrophages which originate from tissue-residing precursors or circulating monocytes extravasate into other tissues under the influence of many kinds of cytokines such as the colony stimulating factors (CSFs) and CCL2. Macrophage-CSF (M-CSF or CSF1), granulocyte-CSF (G-CSF or CSF2) and granulocyte macrophage-CSF (GM-CSF or CSF3) were first identified as *in vitro* hematopoietic growth factors. Early studies in gene-deficient mice showed that M-CSF maintains several macrophage lineage populations important for tissue homeostasis, G-CSF is involved in the control of neutrophil numbers, and GM-CSF is vital for the maturation of alveolar macrophages and invariant natural killer T cells 20. Monocyte chemotactic protein-1 (MCP-1 or CCL2), is considered an inflammatory chemokine because it contributes to monocyte, memory T cell, and NK cell extravasation through vascular endothelium. MCP-1 is overexpressed in triple-negative breast cancers and drives cancer invasiveness and metastasis 21. The above cytokines have been shown to be associated with poor prognosis of TNBC, but there is no clear difference for them. We have an in-depth study for M-CSF in TNBC because M-CSF was the only cytokine of them mentioned in Figure 4B.

Macrophage migration is vital in many biological processes, such as innate immunity, tumor formation and development. Several studies have demonstrated that the change in F-actin content played an important role in the migration of macrophages 22. However, the specific mechanisms by M-CSF mediate the formation of F-actin-rich membrane protrusions and macrophage migrations are not fully understood. Recent evidence indicates that all M-CSF effects are only mediated by the cell surface receptor tyrosine kinase, CSF-1R, and the critical components of CSF-1R downstream molecules, small Rho GTPases Rho, Rac and Cdc42, are closely related with F-actin assembly 23. In addition, the Rho GTPase-dependent assembly of F-actin has been shown to be mediated by members of the Wiskott-Aldrich Syndrome protein (WASP) family of proteins 24. Both WASP family verprolin-homologous (WAVE2) and Abelson kinase interactor 1 (Abi1) were recruited and necessary for the formation of F-actin protrusions in response to M-CSF secretion 25. Although the migration of macrophage has its specificity, the M-CSF/CSF-1R/Rho GTPase/WAVE2-Abi1 complex pathway might be the crucial signal pathway in the skeleton and migration of macrophages which were stimulated by TNBC cells CM.

Classified as part of the mononuclear phagocytic system (MPS), macrophages are a group of terminally differentiated cells that play a critical role in tissue homeostasis, inflammation, and protection against pathogenic infections. As reported previously, the presence of TAM was associated with poor clinical outcome in patients with glioma, cholangiocarcinoma, ovarian, breast cancer, and Hodgkin's lymphoma 26-27. TAMs were involved in an indirect mechanism of promoting tumorigenesis, tumor metastasis and tumor neovascularization along with the tumor-derived angiogenic factors 28. There is strong clinical evidence that high density of macrophages in tumor tissues correlates with poor prognosis of TNBC and high risk of metastasis 29-34. Therefore, TAMs have become an increasingly attractive therapeutic target of cancer immunotherapy. In this study, we not only confirmed the negative correlation between M-CSF secretion and ER expression, but also verified the function of M-CSF on cell cytoskeleton rearrangement promotion and macrophages recruitment in TNBC cells. Hence, M-CSF was the important reason for the malignancy of TNBC and was expected to be a biomarker for poor prognosis of breast cancer.

## Supplementary Material

Supplementary figures and tables.

## Abbreviations

TNBC: triple negative breast cancer
nTNBC: non triple negative breast cancer
M-CSF(CSF1): macrophage colony-stimulating factor
TME: tumor-microenvironment
TAMs: tumor-associated macrophages
ER: estrogen receptor
IOD: Integrated Optical Density
DMEM: Dulbecco^'^s modified Eagle^'^s
RPMI-1640: Roswell Park Memorial Institute-1640
FBS: fetal bovine serum
CM: conditioned medium
MCF-7 cell conditioned medium: M-CM
MDA-CM: MDA-MB-231 cell conditioned medium
H-CM: HCC1937 cell conditioned medium
PBS: phosphate buffer saline
BCA: bicinchoninic acid
MMP-9: matrix metalloprotein-9
ICAM: intercellular cell adhesion molecule
ITGAM: Integrin alpha M
F-actin: filamentous actin
MPS: mononuclear phagocytic system
CSC: cancer stem cell
WASP: Wiskott-Aldrich Syndrome protein
