# Supplementary material for: Macrophage Colony-stimulating Factor Mediates the Recruitment of Macrophages in Triple negative Breast Cancer
Source: Int J Biol Sci. 2019 Nov 8;15(13):2859–71. doi: 10.7150/ijbs.39063 (PMC6909971; doi:10.7150/ijbs.39063)
Supplement: Supplementary file 1 — Supplementary figures and tables. [file ijbsv15p2859s1.pdf]

**Table S1.** The primers used for quantitative polymerase chain reaction.

| Gene | Prime sequence (F 5'-3') | Prime sequence (R 5'-3') |
|---|---|---|
| Icam | CTGAAAGATGAGCTCGAGAGTG | AAACGAATACACGGTGATGGTA |
| Itgam | GAGCATCAATAGCCAGCCTCAGTG | CCAACAGCCAGGTCCATCAAGC |
| Capza1 | GAAGTATTCAATGATGTCCGGC | ATCGTAGCCTTCTATCTTCACG |
| Cfl1 | CAGAAGAAGTGAAGAAACGCAA | AGGTTGCATCATAGAGTGCATA |
| Gapdh | CGGCAAATTCAACGGCACAG | GTTAGTGGGGTCTCGCTCCT |
